# Editorial – Patient perspectives on new therapies for genetic diseases

**DOI:** 10.1515/medgen-2025-2018

**Published:** 2025-07-17

**Authors:** Miriam Elbracht, Christiane Zweier, Ilona Krey-Grauert

**Affiliations:** Uniklinik RWTH Aachen Center for Human Genetics and Genomic Medicine Pauwelsstr. 30 52074 Aachen Germany; Universiy Hospital and University of Bern Department of Human Genetics, Center for Precision Medicine (BCPM) Freiburgstrasse 15 3010 Bern Switzerland; University of Leipzig Medical Center Leipzig Institute of Human Genetics Philipp-Rosenthal-Str. 55 04103 Leipzig Germany

The efforts and technical developments in the last decades have not only increased the diagnostic rate and knowledge of molecular mechanisms in genetic diseases but also translation of these findings into research and application of new therapies. The term advanced therapy medicinal products is divided into gene therapeutics (including CAR T-cell therapies), (somatic) cell therapeutics and tissue-engineered products (TEPs; biotechnologically processed tissue products). As of June 2024, 15 gene therapeutics, two cell therapeutics and two TEPs were authorised in the EU. As of May 2024, there were also 151 authorised medicinal products for rare diseases (orphan drugs) and a further 88 medicinal products that had orphan drug status, but which expired after 10 years of authorisation as prescribed. In most cases, the substances are still available, and new orphan drug therapies are constantly being developed. The total number of ongoing projects in this area is currently estimated to be between 2500 and 3000.

This means that new drugs are increasingly reaching our patients from human genetics outpatient clinics and inpatient clinical care. Evaluating the success of individual therapies remains an ongoing task. Particularly in the case of rare genetic diseases, it will take time before a critical number of patients are recruited and followed over a longer treatment period. Although personal experience reports and individual case descriptions will never take on the significance of larger cohort analyses in the objective cost-benefit assessment of new therapies, they can nevertheless provide a personal impression and an idea of the concrete everyday application.

In the following, three individual patient reports are presented in a short interview format. These reports present personal experiences of individual patients/parents, respectively, obtained with a short questionnaire. They are not acting on behalf of pharma industry, patient organisations or anyone else, would like to stay anonymous and are personally known to the coordinators of this journal’s issue. Their reports were provided in German and translated to English with deepL and by manual revision of the coordinators.

## 1. Cystic fibrosis (CF, Mucoviscidosis) – ORPHA:586

Although Cystic fibrosis (CF) is a rare autosomal recessive disease it represents the most common genetic disorder among Caucasians with a prevalence of 1/5000 in Europe and an average in the general population of 1/9000. The abnormal function of the cellular chlorid channel encoded by the *CFTR* gene leads to viscous mucous secretion and various disease manifestations with leading problems in the respiratory and gastrointestinal tract. By using high-throughput-screening, new compounds for the treatment of CF have been identified. These CFTR modulators revolutionised the therapy for most patients with CF, leading to a significant increase in life quality and expectancy. There are two main groups of CFTR modulators in particular. Potentiators (first potentiator: ivacaftor) increase the probability of the channel opening and correctors (first corrector lumacaftor) that bring the protein to the cell surface at the site of action. Most patients benefit from combination therapies but still access to these new therapies also varies greatly from country to country. In addition, there is a small number of patients who, due to their genetic variants (e. g. premature stop codons), cannot benefit from CFTR modulators. Therefore, continuous improvements and new developments are still necessary (for review, see(1)).

### Report from an adult individual with cystic fibrosis, treated by Kaftrio

**Please tell us more about yourself and your diagnosis (age, gender, diagnosis, genetic findings and age at diagnosis)**

I am 41 years old, female and have cystic fibrosis. Fortunately for me, I was already tested positive for cystic fibrosis during the newborn screening, although this was not yet standardised in 1982. The diagnosis was confirmed 10 weeks later with a sweat test. Genetic testing was not yet done at that time. This was only done 10 years later as part of my family’s fertility counselling. The genotype analysis carried out diagnosed me with the most common CF mutation Delta F508 homozygous.

**Can you briefly describe a typical day before you started using the new drug therapy?**

Despite a still harmless course, the amount of therapy has steadily increased over the years. Before Kaftrio, I needed about 1.5 hours in the morning to clear my lungs of secretions through inhalations and respiratory therapy. Inhaling antibiotics every day for decades was part of my daily routine. During the day, I also had physiotherapy appointments, exercise to strengthen my lungs and general fitness and occasionally an inhalation at lunchtime, depending on which antibiotic or medication needed to be inhaled or what my condition was like. In the evening, I needed another 1–1.5 hours to clear my lungs. Despite these measures, my body was often exhausted from coughing so much and I needed to rest several times a day. In addition to looking after my son, I was only able to work part-time one day a week.

**What medication are you taking, in what dosage and for how long?**

I have been taking the gene modulator Kaftrio (in America Trikafta, active ingredients: ivacaftor, tezacaftor and elexacaftor) since September 2020. I started with the normal dose of two tablets in the morning and one in the evening. After a brief interruption due to side effects in April 2021, I halved the dosage. I now only take one tablet of Kaftrio in the morning, which is even less than half the standard dosage. This dosage is enough for me to keep my lungs almost free of secretions. I only increase the dosage for a short period if necessary.

**Can you describe a typical daily routine now that you are taking the medication? What has changed for you?**

I still usually inhale twice a day, but only with saline solution (NaCl) and only for 10–20 minutes. I no longer need breathing therapy every day, but only every fortnight with my physiotherapist and acutely when I have an infection. There is no more constant coughing, so the body has more energy available. The increased energy and the enormous amount of time I’ve gained allow me to work half-time. I find it a particular luxury to be able to reduce or skip inhalation without any direct loss of lung function.

**Do you notice any side effects from taking the medication, do you have any other critical comments or concerns about the new therapy?**

I have experienced various minor and major side effects. However, the benefits of Kaftrio outweigh them for me. When Kaftrio was launched, one dosage was generally chosen for all adults. This does not seem appropriate to me and should be reconsidered from the outset. In my opinion, the dosage should be adjusted depending on weight, lung function and gender. A more variable dosage option than at present would be desirable and would probably reduce some side effects.

**Finally, please let us know what is particularly important for you to address with regard to the new therapy.**

The cost of Kaftrio must be reduced as quickly as possible so that people with cystic fibrosis in poorer countries can also benefit from the modulators. Not all people with cystic fibrosis can benefit from Kaftrio or other gene modulators due to their mutation. The effects of Kaftrio on the psyche have not yet been clarified. The offer of psychological counselling is desirable here.

## 2. GRIN2B-related neurodevelopmental disorder – ORPHA:589547

*GRIN2B*-related neurodevelopmental disorder is a rare autosomal dominant disease characterized by mild to profound developmental delay / intellectual disability (DD/ID). Muscle tone abnormalities (spasticity and/or hypotonia, occasionally associated with feeding difficulties), as well as epilepsy and autism spectrum disorder (ASD) / behavioral issues, are common. Other infantile- or childhood-onset findings include microcephaly; dystonic, dyskinetic, or choreiform movement disorder; and/or cortical visual impairment. Brain MRI reveals a malformation of cortical development in a minority of affected individuals. To date, a few 100 individuals with *GRIN2B*-related neurodevelopmental disorder are published. In the global GRIN Registry are around 300 individuals with a pathogenic SNV or CNV in *GRIN2B* registered. First genotype-phenotype analysis show a milder phenotype in individuals with null variants compared to missense variants. Even though the clinical presentation is mostly overlapping, the missense variants can be functionally tested and separated by six parameters, classified as “Gain-of-function” (GoF) or “Loss-of-function” (LoF) of the NMDA-receptor (or indeterminant in case of conflicting results) (2). Knowing the functional effect on the NMDAR is of utmost importance for potential precision medicine approaches. In theory, a GoF can be treated with a NMDAR blocker such as memantine or radiprodil. In case of a LoF, receptor agonists (L-serine, spermidine) are conceivable treatment options. For spermidine no published knowledge is available, whereas the positive effect of L-serine is shown in two publications, a retrospective, multicenter, observational analysis and a phase 2A clinical study (3, 4).

### Report from a father about his son who has a GRIN2B-related neurodevelopmental disorder, treated by L-serine and spermidine

**Please tell us more about your son (age, gender, diagnosis, human genetic findings and age at diagnosis).**

My son is now 5 years old and has a pathogenic *de novo* null variant in *GRIN2B*. He was diagnosed in the beginning of 2022 at the age of 2.5 years. Additionally, he has a coeliac disease (diagnosed in 2023). Since then, we have been feeding our son a strictly gluten-free diet.

**Can you briefly describe the therapy with L-serine?**

Our son takes L-serine 3 times a day. The dose is now 8 mg per day. He also takes 3 mg spermidine per day (single dose). The administration varies from easy to difficult and depends on his current condition (i.e. whether he is in a “good” mood or not). Administration is successful in 95 % of cases. Milk is used for administration, as in our opinion it masks best the taste of L-serine and spermidine. The spermidine is administered in the morning by mixing the L-serine and spermidine with cocoa powder and then administering it with a small amount of milk. In addition to the gluten-free diet, the medication is tedious in the long term, especially as there are phases in which it can only be administered with considerable “persuasion”.

**Can you describe a typical day now since the use of L-serine and spermidine? What has changed for you?**

This is one of our biggest uncertainties. We started with spermidine nearly at the same time as we switched to a gluten-free diet (we started taking L-serine long before we were diagnosed with coeliac disease), it is difficult for us to say which positive effect is due to which cause (L-serine, spermidine or “just” the gluten-free diet). We assume that it is a combination of all three factors. In any case, our son has made enormous progress since mid–2023, such as riding a balance bike, walking, language development (forming complete sentences, speaking on his own initiative, easy conversations, etc.), weight gain, diaper-free day and night, almost independent stair climbing, strength and coordination as well as a 50-piece puzzles.

**Do you notice any side effects from taking L-serine or spermidine, do you have any other critical comments or concerns about the new therapy?**

We have not noticed any side effects.

**Finally, please let us know what is particularly important for you to address regarding the new therapy.**

Further research to find a therapy that compensates or ideally balances out the deficits (physical and above all cognitive) caused by the genetic defect.

## 3. Spinal muscular atrophy (SMA) – ORPHA:83330

Currently, three therapeutics for SMA are admitted in Germany. They are either based on splicing modulation to increase the amount of functioning SMN2 protein (Nusinersen (Spinraza®) since 2017, Risdiplam (Evrysdi®) since 2021) or on introducing intact SMN1 into the respective cells in terms of gene replacement therapy (Onasemnogene Abeparvovec (Zolgensma®) since 2021). Due to the availability of targeted therapies, SMA has been implemented in German newborn screening in a pilot project since 2018 and generally since 2021 (5). While these therapies show significant effects in children and clearly prolong lifespan (though not being curative), efficacy in adults is discussed more controversely. These developments also lead to questions/discussions regarding the enormous financial costs but also costs to society by withholding treatments and disadvantaging some individuals (6). Therefore, patient related outcome measures such as health-related quality of life of affected individuals and/or caregivers become more and more important (e.g.(7–9)).

### Report of an adult individual with SMA3, treated by Spinraza®

**Please tell us about yourself and your diagnosis (age and age at diagnosis, gender, genetic findings)**

I am 25 years old, female and have spinal muscular atrophy type 3. I first showed signs of the disease in infancy, but the diagnosis was only made when I was 8 years old and confirmed with genetic testing.

**Can you briefly describe a typical day before you started using the new drug therapy?**

I had a fairly mild course of the disease in my childhood and adolescence, but my condition steadily worsened until I started therapy at the age of 18. In the time shortly before I started the drug therapy, I was already quite severely restricted, although I was still able to walk. Though I was already living on my own, I was dependent on the help of family members for almost all trips outside the house, as I could only walk about 100 metres without feeling severe exhaustion and instability. I could no longer go shopping at the supermarket without needing an extended break to sit down halfway through. I also fell down a lot and had problems getting up from the floor.

**Which medication are you taking, in what dosage and for how long?**

I have been taking Spinraza, also known as Nusinersen, from Biogen since September 2017. This is administered to me intrathecally, i.e. by lumbar puncture, every 4 months.

**Can you describe a typical daily routine now that you are taking the medication? What has changed for you?**

My strength and fitness have improved enormously since starting therapy with Spinraza. I am much more independent (also because I now have a car that has been converted for me) and can manage my everyday life on my own. I can now do the shopping without any problems, as well as short walks with my dog. On particularly good days, I can now even manage walks of several kilometres (4.5 kilometres was my maximum so far) and I am much more stable again and fall down less often. It is also much easier for me to get up again.

**Do you notice any side effects from taking the medication, do you have any other critical comments or concerns about the new therapy?**

I have not experienced any side effects from the drug Spinraza in the years since starting therapy. Only the administration by lumbar puncture often has side effects such as back pain or headaches, but these are negligible for me as it is only three times per year. I would only wish for a higher dosage of the medication or adjusted therapy intervals, as there is usually a noticeable drop in energy and performance a few weeks before the next dose.

**Finally, please let us know what is particularly important to you about the new therapy.**

I am excited about Spinraza and I am convinced that it has saved me from a life in a wheelchair. However, the drug is extremely expensive and the treatment is not covered by health insurance in every country, which is why the therapy is not available to everyone.

## References

[1] Taylor-Cousar JL, Robinson PD, Shteinberg M, Downey DG (2023). CFTR modulator therapy: transforming the landscape of clinical care in cystic fibrosis. Lancet.

[2] Myers SJ, Yuan H, Perszyk RE, Zhang J, Kim S, Nocilla KA (2023). Classification of missense variants in the N-methyl-d-aspartate receptor GRIN gene family as gain- or loss-of-function. Hum Mol Genet.

[3] Julia-Palacios N, Olivella M, Sigatullina Bondarenko M, Ibanez-Mico S, Munoz-Cabello B, Alonso-Luengo O (2024). L-serine treatment in patients with GRIN-related encephalopathy: a phase 2A, non-randomized study. 147(5).

[4] Krey I, von Spiczak S, Johannesen KM, Hikel C, Kurlemann G, Muhle H (2022). L-Serine Treatment is Associated with Improvements in Behavior, EEG, and Seizure Frequency in Individuals with GRIN-Related Disorders Due to Null Variants. Neurotherapeutics.

[5] Muller-Felber W, Blaschek A, Schwartz O, Glaser D, Nennstiel U, Brockow I (2023). Newbornscreening SMA – From Pilot Project to Nationwide Screening in Germany. J Neuromuscul Dis.

[6] Yeo CJJ, Simmons Z, De Vivo DC, Darras BT (2022). Ethical Perspectives on Treatment Options with Spinal Muscular Atrophy Patients. Ann Neurol.

[7] Kolbel H, Modler L, Blaschek A, Schara-Schmidt U, Vill K, Schwartz O (2022). Parental Burden and Quality of Life in 5q-SMA Diagnosed by Newborn Screening. Children.

[8] Patel A, Toro W, Bourke S, Oluboyede Y, Barbier S, Bogoeva N (2024). Treatment preferences in spinal muscular atrophy: A swing weighting study for caregivers of patients with SMA types 1 and 2. PLoS One.

[9] Thimm A, Brakemeier S, Kizina K, Munoz Rosales J, Stolte B, Totzeck A (2021). Assessment of Health-Related Quality of Life in Adult Spinal Muscular Atrophy Under Nusinersen Treatment-A Pilot Study. Front Neurol.

